# Gender Biases in Male Infertility and Its Impact on Women: A Qualitative Exploration

**DOI:** 10.1155/nrp/8103777

**Published:** 2025-05-24

**Authors:** Mehrdad Abdullahzadeh, Zohreh Vanaki, Eesa Mohammadi, Jamileh Mohtashami

**Affiliations:** ^1^Department of Nursing, Faculty of Medical Sciences, Tarbiat Modares University, Tehran, Iran; ^2^Department of Psychiatric Nursing, School of Nursing and Midwifery, Shahid Beheshti University of Medical Sciences, Tehran, Iran

**Keywords:** gender biases, healthcare providers, male infertility treatment, nursing, sociocultural factors, women's perception

## Abstract

**Background:** Infertility affects millions worldwide, and gender biases can shape perceptions of both infertility and treatment, ultimately influencing the quality of care.

**Aim:** This study explores gender biases in male infertility and their impact on affected women.

**Methods:** Conducted in Iran between June and November 2023 by nurse researchers, this qualitative study involved in-depth interviews with six couples whose male partners had primary infertility, a fertility lawyer, and a midwife. The collected data were analyzed using Graneheim and Lundman's inductive content analysis method.

**Results:** The central theme of the findings is “Female Perception of Male Infertility,” guided by three categories: the feminization of infertility, the weight of male infertility treatment on female shoulders, and the pressure of masculinity on women.

**Conclusion:** The findings highlight the existing gender biases regarding male infertility and its treatment and how they affect women with infertility. It is recommended that health policymakers and healthcare providers provide culturally sensitive care and support to reduce the stigma associated with male infertility that affects women and address gender biases regarding male infertility.

## 1. Introduction

Infertility is a complex medical condition that can significantly affect the reproductive health of both men and women. Estimates indicate that approximately one in every six individuals of reproductive age worldwide will experience infertility at some point in their lives [1]. Although biological factors can lead to infertility, it is also affected by psychological, social, economic, and care-related influences [2]. These factors affect both genders; however, societal attitudes toward infertility can vary between men and women [3]. The stereotypes surrounding male virility and fertility can create feelings of inadequacy, blame, and distress in women with infertile partners [4]. In many conservative cultures, women are often held responsible for infertility, which can lead to guilt, shame, and isolation. In addition, women may face accusations from their partners regarding their ability to conceive, even when the infertility originates solely from the male partner [5]. Conversely, infertile men may be disregarded as not being involved in the reproductive process, leading to feelings of neglect and stigma [6].

## 2. Background

Couples struggling with infertility often face complex and emotionally challenging situations. The inability to conceive can cause feelings of sadness, frustration, and disappointment, leading to stress and anxiety [7]. This journey may make the couple feel isolated and alone. Fertility service organizations consider sociocultural structures to be one of the main drivers of pressure on men and women struggling with infertility [8]. In some traditional cultures, women are often held responsible for raising children whereas men are responsible for carrying on the family lineage [9]. As a result, if a man is infertile, women may be blamed even when the infertility of their husbands has been confirmed. This perception can lead to several problems for women, such as societal pressure, lack of support, and discriminatory treatment [10].

Infertility is a complex issue affecting men and women. However, studies have shown that when men are diagnosed with infertility, women tend to react differently. Women may experience disbelief, denial, intense fear, and shock [6, 11]. This information can be particularly overwhelming for women as they try to imagine how male infertility will affect their lives and relationships with their husbands [12]. Fear that they may not be able to lead a calm everyday life and that their husbands may not be the same can be a significant factor causing anxiety in women. These emotions can lead to insecurity and fear as women contemplate the impact of male infertility on their lives and marital stability [13]. Male infertility can cause mutual pain for couples, regardless of the cause. However, it can be particularly challenging for women to overcome, and it can have long-lasting effects [14].

## 3. The Study

Male infertility is a multifaceted and emotionally charged issue that can have a profound impact on the lives of both men and women [15]. Although infertility is commonly believed to be a problem that only women face, male infertility is also a significant issue that affects millions of couples worldwide [16]. Despite this, there remains a lack of understanding about the societal challenges that women with infertile male partners encounter [17]. Recent studies have begun to explore the experiences of women living with male infertility challenges. Nevertheless, there remains a gap in research exploring gender perceptions associated with male infertility [18–20]. In this context, this qualitative study explored gender biases in male infertility and their impact on affected women.

## 4. Methods

### 4.1. Design

We conducted this qualitative descriptive study using a critical approach. Our research was based on the nursing metaparadigm, which emphasizes the importance of the individual and their health, environment, and the role of nursing in providing care [21]. Our manuscript adheres to the requirements for reporting qualitative research (COREQ) [22], and Supporting Table 1 presents the COREQ checklist. 

### 4.2. Study Setting and Recruitment

The study was conducted at a fertility clinic in Isfahan, Iran, between June and November 2023. We included men with primary infertility, women with infertile male partners, and staff members who worked with couples struggling with male infertility. To ensure a diverse sample, the researchers used a purposive sampling method that included individuals with different types of male infertility, age ranges, treatment methods, and geographical locations in Iran. Both male and female participants had to meet specific eligibility criteria, such as receiving confirmation of male infertility from a fertility specialist, having male infertility related only to the male and not a female issue, and not having a history of mental illness, psychotropic drug use, neuropsychiatric drug use, or drug addiction in the past 12 months, as these factors are known to affect men's reproductive health and were, therefore, excluded from the study [23]. Medical staff members who worked directly with couples struggling with male infertility were eligible.

### 4.3. Data Collection

The research team's first author was a Ph.D. candidate and a male registered nurse who was not involved in the study participants' care. The author personally invited eligible participants by providing an invitation letter outlining the study's purpose and emphasizing that participation was voluntary and confidential. Participants who agreed to participate were required to sign a consent form. If an additional person joined the conversation, they were also required to provide consent. The interviews were conducted in a private room at the fertility clinic and were semistructured, focusing on male infertility and gender perceptions associated with male infertility and its treatment. The primary participants in the study were men although the study population consisted of couples. Interviews were conducted mainly with men, but in some cases, spouses requested to be present and contributed to the discussion. These instances were noted, but they did not shift the primary focus. The intent was to interview the men, with any spouse participation considered a natural interaction rather than a formal inclusion of both partners. During the interviews, the participants were asked about their experiences with male infertility, including how gendered perceptions related to male infertility and its treatment affect women with male infertile partners. Additional questions, such as “Can you tell me more?” and “What do you mean?” were asked to clarify the answers provided by the participants. The interviews lasted 20–90 min and were recorded digitally and transcribed verbatim after each session. Participants spoke in Farsi during the discussions, and the first author provided English translations. The translations were reviewed and backtranslated to Farsi to ensure accuracy. Partners were present for the three participants according to their wishes and were active in the discussion. At the end of the interview, the interviewer asked, “Is there anything you would like to add?” During the interviews, the interviewer spoke with six couples. These couples participated in the study two by two. Medical staff were also interviewed to share their experiences with couples experiencing male infertility. In this study, we included medical staff members who were directly involved in caring for, counseling, or treating couples with male infertility. To ensure that participants' professional experiences were appropriately framed, we used semistructured interview guides and encouraged reflective practices. While their professional backgrounds naturally influenced their perspectives, we aimed to minimize bias through objective questioning techniques and thematic analysis methods. Field notes were taken during and after the interviews. Three participants were interviewed twice for additional data and clarification. The data collection process continued until saturation was reached, meaning that possible codes and categories were thoroughly explored and no new ones could be identified. Therefore, the collected data can be considered comprehensive and reflective of the phenomenon under investigation.

### 4.4. Data Analysis

The data analysis employed the conventional qualitative content analysis techniques that Graneheim and Lundman described [24]. The process involved several steps, beginning with the researchers reading each interview transcript multiple times to thoroughly understand each participant's story. The researchers then identified meaning units such as words, sentences, and paragraphs relevant to the study's aim. These meaning units were condensed to shorten the text while maintaining its content. Each condensed meaning unit was assigned a code to summarize its content. The codes were then grouped to form subcategories and categories. Categories and central themes were derived based on interpretations of meaning expressed in the classes. The text included citations, clarifications, and quotations to exemplify the results. The analysis was performed manually using the cut-and-paste method. During the study, the nurse authors held critical meetings to reflect on and review the interpretations of the findings. The research group had diverse backgrounds, including clinical and reproductive healthcare counseling and qualitative research experience.

### 4.5. Rigor and Reflexivity

The purpose of this study was to ensure that the results would be reliable and trustworthy. To achieve this, various measures were implemented, including adhering to Schwandt et al. [25]. The study invited an experienced professor as an external researcher to review the anonymized transcripts. Every step of the research process was documented with utmost attention to detail to ensure that the findings could be easily transferred and replicated in similar studies. The participants' words were accurately recounted without alteration or modification, and transcripts were returned to them for comment and correction to maintain authenticity. The research team also emphasized establishing long-term engagement with the participants throughout the research process, creating a conducive environment that encouraged open and honest feedback. This allowed for a more robust and comprehensive analysis of the findings. Finally, the team made a conscious effort to present the information as engaging and informative, ensuring that the participants' contributions were represented accurately and valued.

### 4.6. Ethical Considerations

This study (Ethics no: IR.MODARES.REC.1401.212) was approved by the Ethics Committee of Tarbiat Modares University. Our interviewer explained the purpose and duration of the study, addressed any concerns, and provided written informed consent forms to the participants. In case of any need, we also offered psychiatric consultations and ensured anonymity by assigning unique secret code numbers to each participant. In this study, data storage was carefully managed to ensure security and integrity. We used secure local databases and encrypted methods to store the collected information. In addition, we implemented various security measures, including encryption, access control, and regular audits, to protect sensitive data and prevent unauthorized access. All the procedures conducted in this study, which involved human participants, complied with the institutional and national research committee's ethical standards, the 2013 Helsinki Declaration, and comparable ethical standards.

## 5. Findings

### 5.1. Characteristics of the Participants

The study involved 14 participants: six couples with partners having primary male infertility, a fertility lawyer, and a midwife.  Table 1 lists the participants' demographic characteristics.

### 5.2. Qualitative Findings

**Figure 1:**
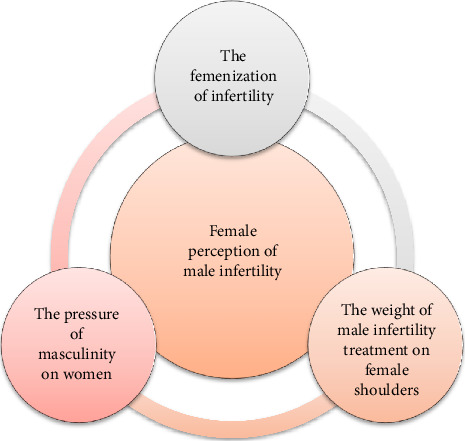
Model illustrating the main findings from gendered perceptions associated with male infertility and its treatment that affects women who have partners with male infertility.

Based on our data analysis, we identified one central theme and categorized our findings into three categories. To better understand the results, we created an abstract model (Figure 1).

We have also included direct participants' quotes to further illustrate these categories. Tracking and confirmation of findings and condensed codes are presented in Supporting Table 2.

#### 5.2.1. Female Perception of Male Infertility

The main topic of the conversation is “Female Perception of Male Infertility,” which covers the underlying principles of various categories. This theme involves the intricate nature of gendered perceptions associated with male infertility and its treatment that affects women who have partners with male infertility.

##### 5.2.1.1. Feminization of Infertility

Based on the feedback received from the participants, the predominant factors contributing to the feminization of infertility were the gendered perceptions associated with male infertility and its treatment directed toward women whose partners were diagnosed with male infertility. A person recounted personal experiences in the following manner:

*Early on in our marriage, I thought it was my wife's problem because we had no history. Then my wife said, “Oh my God, what stress did you put on me that day because you thought the problem was my fault?” At that time, I kept asking my wife, “Why don't you have children?” Let us go to the doctor; take this medicine every time; maybe it was my misbehavior,” (he says with a sigh). We were crazy; we wanted to have a baby soon. When we went for an ultrasound several times, the doctors did not see any problem with my wife. After they sent me to take a test, the doctor realized my problem (male infertility) was with me. I could not believe it. How can a man with regular sexual activity be infertile?* (Couple 5, Man).

These attitudes and expectations significantly influence the perception and experience of male infertility in such situations. The fertility lawyer mentioned

*In most families, having children is considered more important for women than men. Consequently, women are given priority when treating infertility. This bias exists even among doctors and other medical professionals. It is often assumed that men do not have fertility issues, leading to a lack of understanding of male infertility. It is a common mindset that infertility is a female issue* (Fertility lawyer).

##### 5.2.1.2. Weight of Male Infertility Treatment on Female Shoulders

Participants stated that because of societal norms and expectations, women are burdened with the responsibility of managing male infertility. For example, a midwife said

*Most of our patients were women who sought infertility treatment. Men usually do not think they can have infertility problems, so they blame the woman. Therefore, we start with the woman and test her using various drugs and methods until we rule out any issues. However, if she still does not get pregnant, we think the man might be the cause. Then, we request that he perform a test to discover any difficulties he may face* (Midwife).

According to the participants, men's discomfort in discussing reproductive health often leads to women taking the responsibility of seeking medical help and providing emotional support during diagnosis and treatment. A woman mentioned

*My husband is putting pressure on me, even though he knows that the issue is with him. He repeatedly asked me about my fertility status, as if he were trying to shift all the blame to me. Our relationship is falling apart because of this. He insists that our inability to have children is solely my fault, and the burden is on me. Despite what the doctor has explained—that it is a one-sided problem—he remains unconvinced and uncooperative with treatment* (Couple 2, Wife).

##### 5.2.1.3. Pressure on Women of Masculinity

According to the feedback provided by the participants, the prevailing gendered perceptions associated with male infertility and its treatment toward masculinity had a considerable impact on women who were in a relationship with male partners experiencing infertility issues. For instance, a woman shared her experiences:

*My husband blamed me for our infertility. He ignored the doctor's diagnosis that his sperm had defects and problems. He kept questioning my fertility and making me feel guilty. Everyone sided with him and accused me of being the cause. He refused to acknowledge his role in our infertility* (Couple 1, Wife).

These women are often subjected to immense pressure and expectations, which can further intensify their emotional and psychological distress. Another woman said

*He (my husband) did not trust his doctor's words or seek any treatment. I think our culture influences this. It is easier to point a finger at a woman than a man. This culture hurts both women and men, and it needs to be changed. We must understand that infertility is a human issue that can affect any couple, regardless of gender* (Couple 4, Wife).

## 6. Discussion

This qualitative study explored gender biases in male infertility and their impact on women affected by the same. The study primarily investigates how male infertility affects women, but it also includes men's perspectives to offer context and a deeper understanding of their partners' experiences. By incorporating their voices, the study illustrates how male infertility is perceived within the couple dynamic and how it influences women's emotional and social responses. Furthermore, the narratives from the medical staff enrich the discussion on gender bias by revealing the societal and professional attitudes toward infertility. The qualitative findings indicate that cultural norms and personal experiences shape the perception of male infertility, illustrating the intricate relationship between society and individual emotions. The study's most significant finding is the female-dominant view of male infertility, which provides insight into the prevailing gender perception of this issue. This view intensifies the challenges faced by women with infertile partners. The findings of this study indicate that the widely held belief that infertility is exclusively a female problem can have disproportionate impacts on women whose partners are facing male infertility issues. Turner et al. argued that the feminization of infertility is due to societal perceptions of medicine as a female-focused field and the historical burden placed on women for childbearing and domesticity [26]. According to Bornstein et al., societal attitudes and expectations toward infertility can significantly impact how women with infertile male partners experience male infertility and its treatments [27]. Healthcare providers and policymakers should acknowledge and address the culturally gendered perceptions related to male infertility and its treatment to offer better support and care for couples facing male infertility.

Our findings revealed that society often places an unjust and disproportionate burden on women when treating male infertility. According to the research by Sharma and Shrivastava, women who have partners suffering from male infertility tend to experience a wide range of adverse effects, such as high levels of emotional and mental stress, physical strain, and financial hardship related to male infertility treatments [28]. Ravitsky and Kimmins noted that the societal expectations and attitudes toward male infertility, which often place the sole responsibility of bearing a child on women, only intensify the burden of male infertility treatment on them [29]. Therefore, healthcare providers and policymakers must increase awareness and education to address gender inequality and promote equal responsibility and support for partners struggling with male infertility.

Based on our findings, societal expectations and traditional notions surrounding masculinity can have a significant impact on women who are in relationships with male partners facing infertility challenges. Bolten et al. showed that the gendered stereotypes attached to male virility and fertility can lead to a sense of inadequacy, blame, and distress among women who are unable to conceive with their infertile male partners [4]. As Bueno-Sánchez et al. stated that these stereotypes can create a toxic environment for women with male infertile partners, leading to significant psychological and emotional stress [30]. Therefore, to create more inclusive environments for women facing male infertility challenges, healthcare providers and policymakers must challenge stereotypes, promote open communication, encourage professional help-seeking, and educate the public. This will help couples navigate challenges more efficiently, leading to better mental health outcomes.

### 6.1. Limitations of Work

The findings of this study apply only to a particular group of men and women experiencing male infertility. Therefore, it may only be appropriate to generalize these findings to individuals facing similar health challenges. Communication barriers may prevent some participants from participating in the study, potentially affecting the results. In addition, some participants might need assistance from research assistants for social or cultural reasons that could impact their participation and the study's overall results. Thus, it is crucial to consider these limitations and nuances when interpreting the study outcomes.

### 6.2. Implications for Research, Practice, and Policy

This study indicates potential actions for future research, policy, and practice based on its findings. Researchers can conduct a more extensive survey to determine common and harmful gender stereotypes related to male infertility for women's mental health, examine how culture and society influence these stereotypes, and explore how male infertility affects the marital bond of both men and women. Healthcare providers should offer emotional help to both men and women who have infertility problems, promote honest communication between partners, advise them on managing the stress of infertility, and teach other healthcare providers how to identify and handle gender stereotypes in the diagnosis and treatment of infertility. Policymakers can make policies that support equal rights for men and women in getting infertility care, no matter what the cause is, inform about how infertility has different implications for men and women and how it affects women's mental health and create guidelines for healthcare providers to deal with the gender stereotypes related to male infertility and help women who have infertile male partners.

## 7. Conclusion

Dealing with infertility can be a challenging and emotional journey for both men and women. This study highlights gender biases and societal perceptions of male infertility and its treatment. The feminization of infertility makes the burden even more significant for women with infertile partners and highlights the need for healthcare providers and policymakers to address these gendered perceptions. By recognizing and challenging these biases, healthcare providers can create more inclusive and supportive environments for couples facing male infertility challenges. It is essential to acknowledge the human aspect of infertility and consider the experiences of patients and their partners when providing treatment and care beyond gender. Through increased awareness and education, reproductive health outcomes can be improved for all individuals and couples struggling with infertility. The results of this study can serve as a starting point for future person-centered and equitable infertility visitation policies that consider the impacts on patient wellbeing and how individual feelings of vulnerability and safety may shape their experiences.

## Figures and Tables

**Table 1:** Characteristics of the participants.

Alias	Participant	Age (years)	Being with the spouse (years)	Time trying to conceive (years)	Position	Education
1	Wife	29	5	4	Housewife	Primary school
	Man	30			Freelance	Diploma

2	Wife	39	10	9	Housewife	Associate degree
	Man	37	15	10	Employee	Associate degree

3	Wife	41	18	15	Housewife	Primary school
	Man	47			Worker	High school

4	Wife	30	10	9	Freelance	Associate degree
	Man	34			Freelance	Master's degree

5	Wife	27	10	9	Employee	Diploma
	Man	39			Worker	Primary school

6	Wife	43	11	2	Housewife	Diploma
	Man	38			Freelance	Diploma

			Gender	Marital status		

Midwife		40	Female	Single		Bachelor's degree

Fertility lawyer		33	Female	Marred		Bachelor's degree

## Data Availability

The data that support the findings of this study are available from the corresponding author upon reasonable request.

## Supporting Information

Supporting Information 1 Supplementary Table 1 includes the COREQ checklist, a 32-item framework designed to ensure transparent and rigorous reporting of qualitative studies. It encompasses three main domains: Research Team and Reflexivity, Study Design, and Analysis and Findings. This checklist details aspects such as interviewer qualifications, participant selection, data collection methods, and the clarity of reporting, all aimed at enhancing the quality of research.

Supporting Information 2 Supplementary Table 2 presents condensation codes and categories that reflect perceptions of male infertility. It focuses on cultural beliefs, gender biases, and societal expectations. The table underscores the feminization of infertility, the associated burden on women, and the impact of masculinity on treatment approaches, providing insights into the gendered dynamics of male infertility.
