# Supplementary material for: Gender Biases in Male Infertility and Its Impact on Women: A Qualitative Exploration
Source: Nurs Res Pract. 2025 May 24;2025:8103777. doi: 10.1155/nrp/8103777 (PMC12126270; doi:10.1155/nrp/8103777)
Supplement: Supporting Information 2 — Supplementary Table 2 presents condensation codes and categories that reflect perceptions of male infertility. It focuses on cultural beliefs, gender biases, and societal expectations. The table underscores the feminization of infertility, the associated burden on women, and the impact of masculinity on treatment approaches, providing insights into the gendered dynamics of male infertility. [file 8103777.f2.docx]

**Supplementary 2:** The track and confirmation of findings and codes

| **Condensed codes** | **Categories** | **Theme** |
| --- | --- | --- |
| Emphasis on the popular belief that infertility is a female issue | The feminization of infertility | The Feminine Perception of Male Infertility |
| Paying attention to cultural beliefs about infertility as an obstacle to advancing the treatment process with both couples |  |  |
| Emphasis on the very few visits of men to perform fertility tests before women |  |  |
| Emphasis on the existence of this mindset in the treatment staff that infertility is a female issue |  |  |
| Advancement of women for treatment by understanding the importance of having children for women |  |  |
| A woman's initiative to go to a fertility clinic to understand the problem |  |  |
| A woman taking the initiative to start treatment with the belief that infertility is a female issue |  |  |
| Imposing the pressure of infertility treatment on the woman by the medical center in the form of giving priority to women's examinations | The weight of male infertility treatment on female shoulders |  |
| Emphasis on women's desire to receive donated sperm than to receive donated embryos, despite the man's opposition |  |  |
| Emphasis on imposing the responsibility of men's infertility on women by society, always holding women responsible for infertility |  |  |
| Emphasis on imposing the follow-up of infertility treatment on the woman by the man |  |  |
| Imposing the burden of seeing a psychotherapist on the woman at the treatment center |  |  |
| Therapists doubt the man's infertility after rejecting the woman's infertility |  |  |
| Emphasizing the necessity of creating a culture to face male infertility | The pressure of masculinity on women |  |
| The existence of a cultural view of masculinity as a psychological burden for infertile men |  |  |
| Denying the reality of male infertility by men due to the cultural stereotype of masculinity |  |  |
| The existence of a cultural view of masculinity as a psychological burden for women with infertile husbands |  |  |
| Common belief that men do not have infertility problems |  |  |
| Emphasis on the impact of receiving donated sperm for masculinity identity |  |  |
| Not expressing discomfort with infertility due to public expectation of male identity |  |  |
| Emphasizing the equality of reproductive ability and masculinity identity for men |  |  |
| Emphasis on society's inattention to cultural-social pressures of infertility on men |  |  |
